# Measuring fluid balance in end-stage renal disease with a wearable bioimpedance sensor

**DOI:** 10.1186/s12882-024-03929-9

**Published:** 2025-01-08

**Authors:** Frida Bremnes, Cecilia Montgomery Øien, Jørn Kvaerness, Ellen Andreassen Jaatun, Sigve Nyvik Aas, Terje Saether, Henrik Lund, Solfrid Romundstad

**Affiliations:** 1 Mode Sensors AS, Trondheim, Norway; 2 Department of Nephrology, St. Olavs Hospital, Trondheim, Norway; 3 Department of Internal Medicine, Levanger Hospital, Nord-Trøndelag Health Trust, Levanger, Norway; 4 Department of Clinical and Molecular Medicine, Norwegian University of Science and Technology (NTNU), Trondheim, Norway; 5 Professor Brochs Gate 2, Trondheim, 7030 Norway

**Keywords:** Bioimpedance, Hydration, Kidney failure

## Abstract

**Background:**

Accurate assessment of fluid volume and hydration status is essential in many disease states, including patients with chronic kidney disease. The aim of this study was to investigate the ability of a wearable continuous bioimpedance sensor to detect changes in fluid volume in patients undergoing regular hemodialysis (HD).

**Methods:**

31 patients with end-stage renal disease were enrolled and monitored with a sensor patch (Re:Balans^®^) on the upper back through two consecutive HD sessions and the interdialytic period between. The extracellular resistance R_E_ was calculated from multi-frequency bioimpedance measurements and was hypothesized to correlate with the amount of extracted fluid during dialysis.

**Results:**

Only HD sessions with a positive net fluid extraction were included in the primary analysis. Participants had an increase of 7.5 ± 4.3 Ω (Ohm) in R_E_ during the first HD and 6.2 ± 2.3 Ω during the second HD, and a fluid extraction (ultrafiltration (UF) volume) of 1.5 ± 0.8 L and 1.2 ± 0.6 L, respectively. The relative change in R_E_ during HD correlated strongly with UF volume (*r* = 0.82, *p* < 0.001). During the interdialytic period, the patients had a mean decrease in R_E_ of 6.0 ± 3.5 Ω. Longitudinal changes in R_E_ (%) and body weight (kg) over the entire study period was negatively correlated (*r* = -0.61 *p* < 0.001). Longitudinal changes in blood samples and cardiovascular changes were also in agreement with changes in weight and R_E_.

**Conclusions:**

The results of this clinical investigation indicate that the investigational device is capable of tracking both rapid and gradual changes in hydration status in patients undergoing regular HD.

**Supplementary Information:**

The online version contains supplementary material available at 10.1186/s12882-024-03929-9.

## Background

The regulation of body fluid balance is a key concern in several disease states. Chronic kidney disease (CKD) is one of the diseases where altered hydration is a significant part of the pathophysiology [1]. Fluid accumulation in CKD may lead to peripheral edema, pulmonary edema, and hypertension [2, 3]. Volume overload is an independent predictor of mortality in this patient group [4], and proper management of fluid balance is therefore crucial.

Currently, there is no gold standard for feasible, reliable, and affordable assessments of fluid balance, and the high costs and patient burden related to recurrent hospitalization of patients with fluid management problems are concerning. Body weight is commonly used to assess fluid balance, but is subject to changes unrelated to hydration, such as changes in muscle mass/fat mass, and the content of the gastrointestinal tract [5, 6]. The use of clinical signs and symptoms, blood pressure, ultrasound, and biomarkers also have limitations [7–9].

Several studies point to the potential benefit of bioelectrical impedance analysis (BIA) in fluid management [10–12]. BIA uses resistance to electrical current to estimate the fluid content of the body or a body segment. Moreover, measuring impedance over a wide frequency range, termed bioimpedance spectroscopy (BIS), allows differentiation between extracellular water (ECW) and intracellular water (ICW) [13]. Lately, wearable bioimpedance devices have been developed, enabling continuous monitoring of fluid balance, and thus increasing the potential clinical value of this technology. The successful development of a lightweight non-invasive continuous hydration monitor is likely to have significant benefits for a wide range of patient populations, including kidney disease, heart failure, lymph edema, and patients prone to dehydration due to stomas or reduced sensation of thirst (e.g. elderly individuals). In the particular use case of patients with CKD, continuous fluid monitoring may aid in the adjustments to a patient’s treatment plan, with the goal of reducing the number and severity of adverse events caused by large fluctuations in fluid balance.

In the present clinical investigation, the ability of a new wearable bioimpedance sensor to detect changes in fluid volume was investigated. Patients with end-stage renal disease (ESRD) were monitored every 30 s during two consecutive dialysis sessions and the interdialytic period between. The aim of the investigation was to assess the performance of the sensor, by investigating the correlation between ultrafiltration (UF) volume during dialysis and the measured change in bioimpedance. It was hypothesized that changes in the extracellular resistance R_E_ would correlate with the amount of extracted fluid during dialysis. The sensor’s ability to follow longitudinal fluctuations in fluid balance was also explored.

## Methods

### Study design and participants

This prospective cohort study was conducted in the hemodialysis (HD) units of Levanger Hospital (Levanger) and St. Olavs Hospital (Trondheim) in Norway in May and June 2021. Adult HD patients able to provide informed consent were eligible to participate. Exclusion criteria were acute intercurrent disease and known hypersensitivity to plasters/adhesives. Patients were monitored during two to six HD sessions. Patients were also monitored between dialysis sessions.

**Fig. 1:**
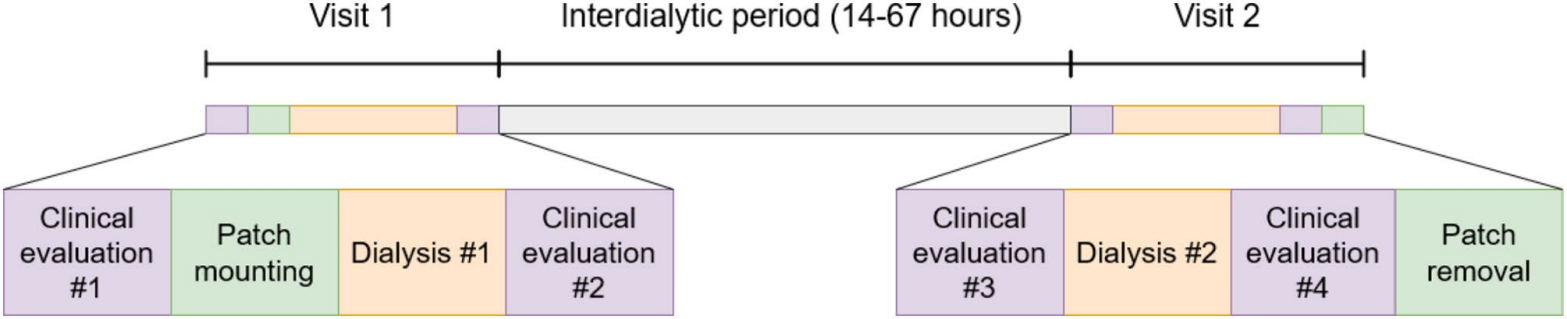
Study design. The clinical evaluation included an assessment of body weight, blood pressure, and a blood sample

The clinical investigation endpoints were intra- and interdialytic changes in R_E_ and R_T_ (explained below), UF volume (fluid extraction during HD), body weight, blood pressure, and blood parameters. The primary outcome measure was the correlation between UF volume and measured bioimpedance change. The study design is presented in Fig. 1.

### Investigational device

**Fig. 2:**
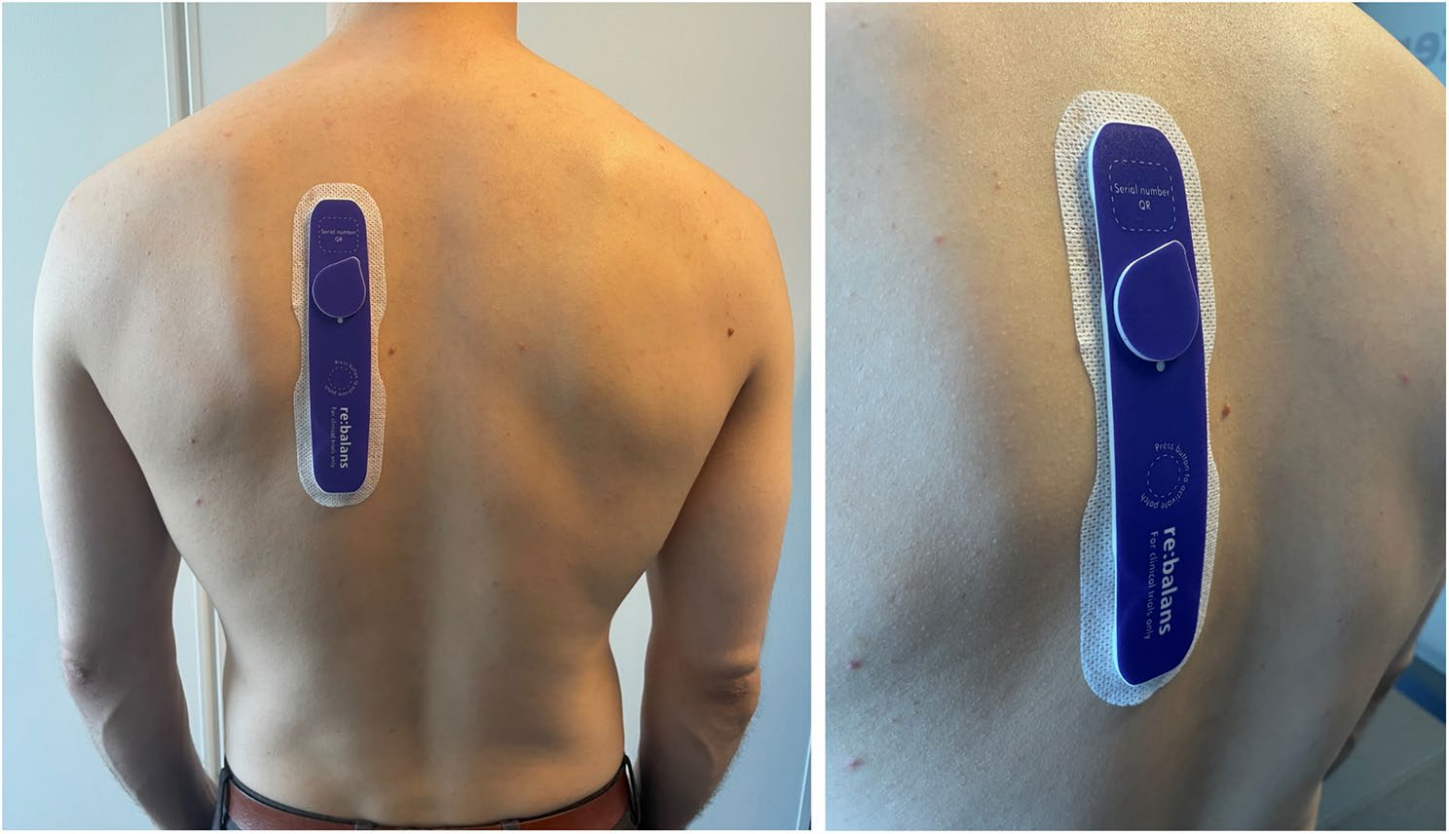
The version of Re:Balans^®^ used in the study

The investigational device (Re:Balans^®^, Mode Sensors AS, www.modesensors.com) is a lightweight (approx. 15 g), wearable non-invasive bioimpedance sensor, with the form-factor of an adhesive patch. The patch consists of a printed circuit board, a coin cell battery, and measuring hydrogel electrodes, all encapsulated by protective die-cut foam and skin adhesive. The version used in the current study is shown in Fig. 2. The sensor is flexible and has four integrated electrodes. The outer electrodes are used for current injection, while the inner electrodes are used for voltage measurements. In this study, measurements were performed at 24 distinct frequencies, ranging from 1 kHz to 313 kHz. Impedance is calculated at each frequency based on the constant injection current and the voltage measured over the inner electrodes. The tissue frequency response is used to estimate extracellular resistance (R_E_) and total resistance (R_T_) for each measurement cycle. R_T_ is expected to reflect both intracellular and extracellular fluid. R_E_ is expected to primarily reflect extracellular fluid. The version used in this clinical investigation performed measurements every 30 s.

In the current study, the application site of the investigational device was the upper back, between the shoulder blades (scapulas), 2–4 cm left or right of the spine. The tissue directly underneath the patch is skin (epidermis, dermis), subcutaneous adipose tissue (SAT), and muscle (m. trapezius, m. rhomboideus major, m. erector spinae). Given the distance between the inner electrodes, the vast majority of the measured impedance is expected to reflect muscle, skin, and adipose tissue [14, 15]. Because a tetrapolar set-up is used (four electrodes) the skin-electrode contact impedance will have a limited impact on the measured impedance [15].

### Hemodialysis equipment

Different dialysis machines were used at the two investigational sites. At site A, the dialysis machine used was 5008 Hemodialysis System (Fresenius Medical Care). At site B, the dialysis machine used was Nikkiso Dbb-Exa (Scan-Med). Blood pressure was measured regularly throughout the dialysis session by the dialysis machine.

### Blood sample analyses

Blood samples were obtained before and after each HD session to assess different markers of kidney failure, electrolytes, and other blood parameters. Blood samples were analyzed for blood urea nitrogen (BUN), creatinine, potassium, sodium, magnesium, glucose, albumin, hemoglobin, leukocytes, and thrombocytes, using an Architect ci8200 autoanalyzer (Abbot Diagnostic, Longford, Ireland). Osmolarity was calculated as (2*Sodium) + Glucose + BUN. Creatinine was used to calculate estimated glomerular filtration rate (eGFR), using the Chronic Kidney Disease Epidemiology Collaboration formula (CKD-EPI) [16].

### Trial registration

The clinical investigation was registered on clinicaltrials.gov (NCT04937478, published 24/06/2021).

### Statistical analyses

Baseline data are expressed as mean ± standard deviation, or mean and range. Absolute and relative changes are presented with mean ± standard deviation. Correlations were investigated using Pearson’s correlation coefficient. A coefficient of 0.2–0.39 was considered weak, 0.4–0.69 was considered moderate, and > 0.7 was considered a strong correlation. A significance level of 0.05 was used for all tests. Statistical analyses were performed using Python and a combination of open-source packages, including NumPy, Pandas, SciPy, and Statsmodels.

Each bioimpedance measurement cycle is subject to a range of quality assurance gates. Only measurements that pass these quality assurance steps are forwarded as valid measurements. Invalid measurements can arise from a variety of sources such as poor contact with the skin, water intrusion, or electromagnetic interference from various sources. In the present study, patches with less than 80% valid measurement cycles were excluded from the analysis.

Due to intake of food and fluid during HD and low UF volumes, it was noted that several of the enrolled participants had no net fluid loss during HD despite having fluid extracted during HD. To investigate the relationship between the amount of extracted fluid and measured bioimpedance (the primary outcome), a criteria of weight loss during HD was applied post hoc. The criterion was implemented to ensure a more homogeneous population with a definitive physiological response to the procedure (net fluid loss).

### Study population

31 subjects were recruited for the investigation. The study was a real-life study with a consecutive sampling strategy. The result of this enrolment strategy was a heterogeneous non-selected population of HD patients with different levels of disease severity and a range of comorbidities. Subject characteristics are summarized in Table 1.

**Table 1:** Subject characteristics

	All participants	Participants included in primary analysis
**Number (men/women)**	31 (25/6)	16 (14/2)
**Age (mean)**	71 (range 48–83)	73 (range 57–83)
**Body mass index (mean kg/m** ^**2**^ **)**	30 (range 22–44)	29 (range 22–35)
**Main diagnosis**		
Nephrosclerosis	13	8
Adult polycystic kidney disease	6	3
Other	12	5
**Comorbidities**		
Cardiac disease	11	5
Lung Disease	3	1
Atrial Hypertension	7	5
Diabetes	15	8
Overweight (25–30)	13	9
Obesity (BMI > 30)	13	5

The subjects underwent either two (*N* = 9), four (*N* = 20), or six (*N* = 2) HD sessions, corresponding to a total of 110 sessions. Each investigational device (patch) was used for two consecutive sessions, and the total number of patches used was therefore 55. Five of the patches had less than 80% valid measurement cycles and were excluded from the analysis (10 excluded HD sessions). In total 8 HD sessions were excluded due to missing metadata (one subject) and an unrelated adverse event (hypotension resulting in two bolus doses of intravenous fluid). In addition, 46 sessions were excluded from the primary analysis due to the criteria of weight loss (> 0 kg) during HD. A total of 46 dialysis sessions from 16 unique subjects were included in the primary analysis. Some analyses required data from two consecutive HD sessions measured by the same sensor, resulting in an additional exclusion of 6 single sessions. The longitudinal analysis required that participants had minimum two HD sessions included, resulting in exclusion of 2 subjects with only one included HD session. All subjects and patch exposures were included in the safety analysis.

## Results

### Intra- and interdialytic changes in bioimpedance

**Fig. 3:**
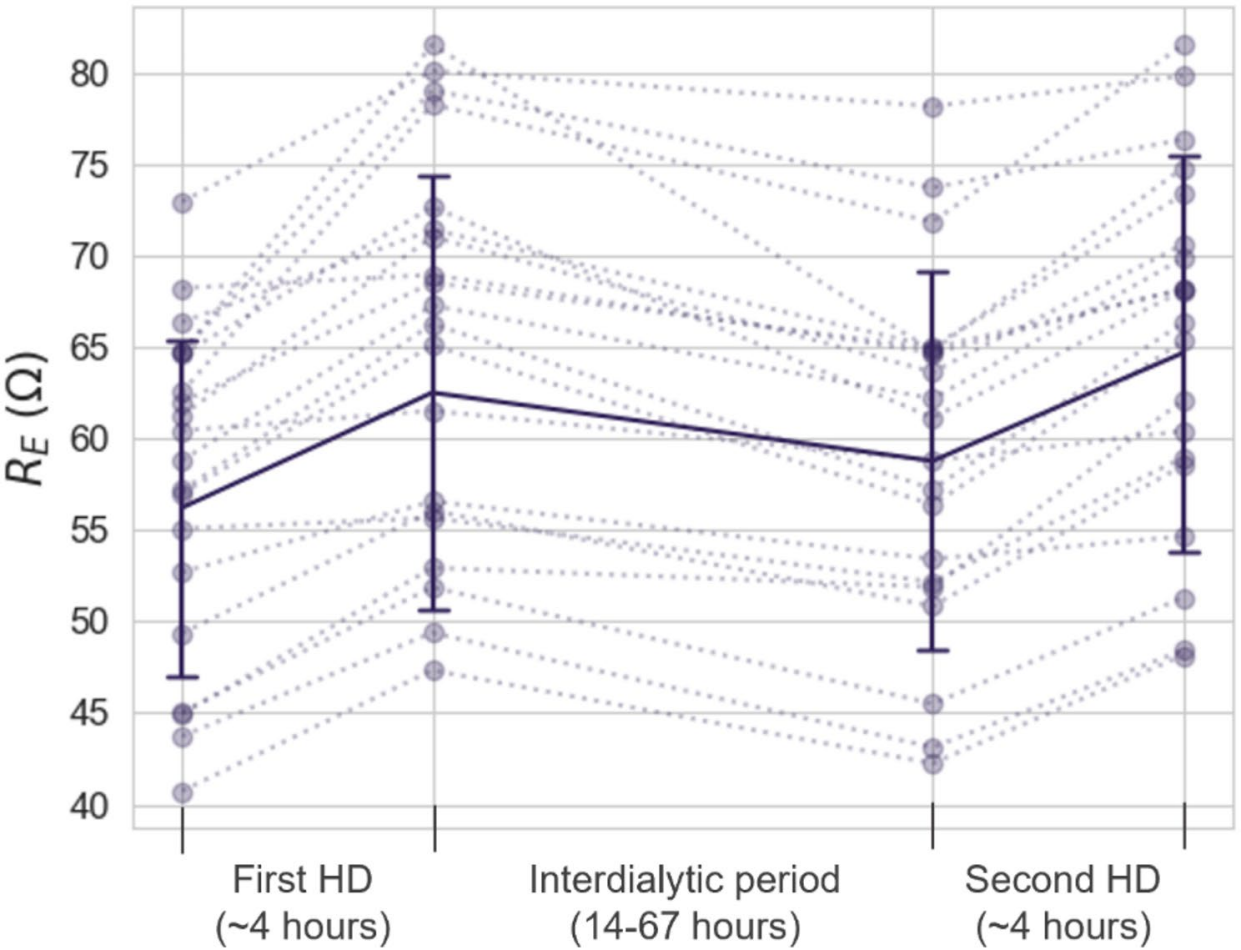
Extracellular resistance (R_E_) measured at four time points (before and after the first HD, before and after second HD). The figure displays both individual plots (stippled lines) and mean values ± SD (solid line)

Figure 3 displays the mean R_E_ at the time points before and after two consecutive dialysis sessions measured by the same investigational device (*N* = 20 pairs). The figure also presents individual values for the same time points. On average, participants had an increase of 7.5 ± 4.3 Ω (ohm) in R_E_ during the first HD, followed by a decrease of -6.0 ± 3.5 Ω during the interdialytic period. During the second HD, R_E_ increased by 6.2 ± 2.3 Ω (all *p* < 0.001). The average UF volume drawn was 1.5 ± 0.8 L for the first HD and 1.2 ± 0.6 L for the second HD. There was a high interpersonal difference in absolute resistance, but the individual participants followed the general pattern of increased resistance during HD and decreased resistance in the interdialytic period. In general, participants were brought to a similar R_E_ value at the end of the two HD sessions, with 2.2 ± 2.3 Ω as the mean absolute difference between the two time points for all 20 HD pairs.

Changes in R_T_ followed the same pattern as R_E_ but were overall less pronounced. On average, R_T_ increased by 5.0 ± 3.3 Ω during the first HD, decreased by 3.2 ± 2.4 Ω during the interdialytic period, and increased by 3.9 ± 1.9 Ω during the second HD (all *p* < 0.001).

### Bioimpedance and UF volume during dialysis

**Fig. 4:**
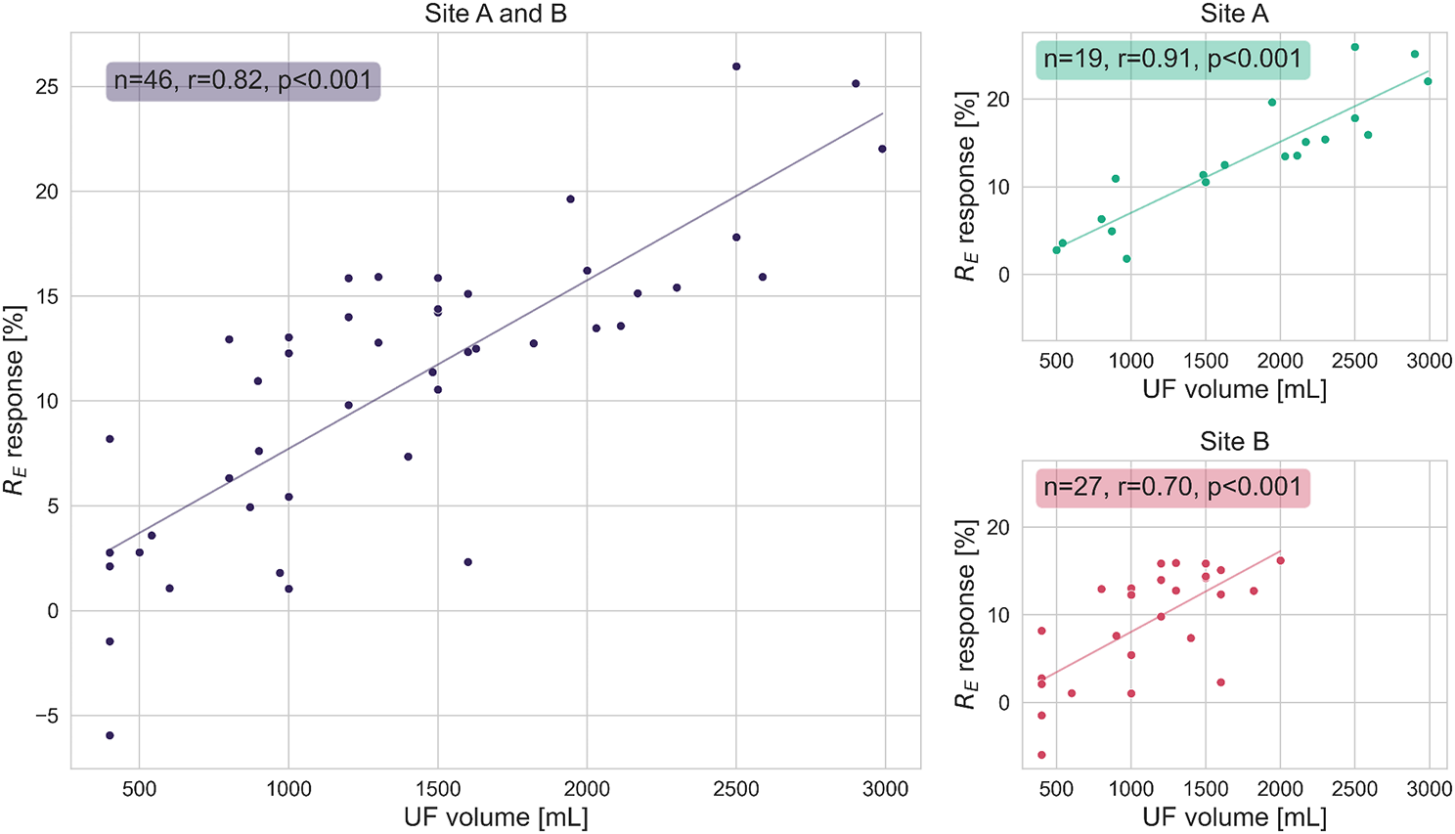
Correlation between UF volume [mL] and R_E_ response [%] during HD. The main plot displays data from all included HD sessions. The subplots display correlations for the individual study sites. The % change in R_E_ is calculated relative to the start of each dialysis session

During HD sessions, a strong correlation between the R_E_ response and UF volume was observed (*r* = 0.82, *p* < 0.001; Fig. 4). When evaluating the sites individually, they both displayed a strong significant correlation between R_E_ and UF volume, but it was noted to be considerably higher for site A (*r* = 0.91, *p* < 0.001) than for site B (*r* = 0.70, *p* < 0.001). The mean UF volume was also higher at site A (1.7 ± 0.8 L at site A, 1.1 ± 0.5 L at site B). The UF volume also correlated with the R_T_ response (*r* = 0.76, *p* < 0.001).

### Intradialytic changes in blood parameters

Blood samples were drawn before and after each HD session. Complete results are presented in Appendix I (Supplementary material). In short, changes in blood parameters were as expected in ESRD patients undergoing an HD session.

The change in R_E_ during dialysis correlated with several blood parameters. The R_E_ response had a moderate positive correlation with the intradialytic change in hemoglobin (*r* = 0.65, *p* < 0.001), and a moderate negative correlation with sodium (*r* = -0.40, *p* < 0.01). There was a weak correlation between the R_E_ response and the intradialytic change in osmolarity (*r* = -0.39, *p* < 0.01), BUN (*r* = -0.32, *p* < 0.05), and glucose (*r* = 0.32, *p* < 0.05).

The R_T_ response had a moderate positive correlation with hemoglobin (*r* = 0.56, *p* < 0.001), and a weak correlation with intradialytic changes in osmolarity (*r* = -0.34, *p* < 0.05), sodium (*r* = -0.32, *p* < 0.05) and BUN (*r* = -0.30, *p* < 0.05). There was no significant correlation between either the R_E_ or R_T_ response and the intradialytic change in leukocytes, thrombocytes, creatinine, magnesium, and eGFR.

### Intradialytic changes in blood pressure

The intradialytic change in blood pressure was calculated as the difference between the first and last blood pressure measurements performed by the dialysis machine during the HD session. There was a weak negative correlation between the R_E_ response and changes in systolic blood pressure (*r* = -0.32, *p* < 0.05). No correlation was found between the changes in diastolic blood pressure and the R_E_ response. These findings were consistent with the relationship between UF volume and changes in systolic blood pressure (*r* = -0.34, *p* < 0.05), and the fact that there was no correlation between UF volume and changes in diastolic blood pressure.

### Longitudinal analysis of fluid status

The participants were subject to up to three monitoring periods measured by different investigational devices. The time between two consecutive monitoring periods was up to 34 days.

To investigate the ability of the investigational device to follow fluctuations in fluid status over a longer period, the measurements performed before and after the participant’s included HD sessions were compared to an individualized baseline. The measurements performed after the first included HD session with complete data was used as the baseline for each subject, and it is assumed that the subject is in an optimal fluid state at this time point. Two of the participants included in the primary analysis were excluded from the longitudinal analysis as they only had one HD session and no baseline could be established. Hence, in total 60 comparisons from 14 unique participants with a variable number of HD sessions were evaluated (*N* = 5 with 2 HD sessions, *N* = 2 with 3 HD sessions, and *N* = 7 with 4 HD sessions). The time between the baseline HD and the last HD varied from 1 to 39 days with an average of 11 days.

To get a more comprehensive overview of the patient’s fluid status, a combination of changes in body weight (kg), blood pressure (mmHg), hemoglobin (g/dL), hematocrit, albumin (g/dL), and accumulation of the waste products BUN (mmol/L) and creatinine (µmol/L) were evaluated and compared to changes in R_E_ (%) and R_T_ (%). As body weight is considered a central marker of short-term changes in fluid status in patients with ESRD, the other parameters were also compared to changes in body weight.

### Changes in body weight

**Fig. 5:**
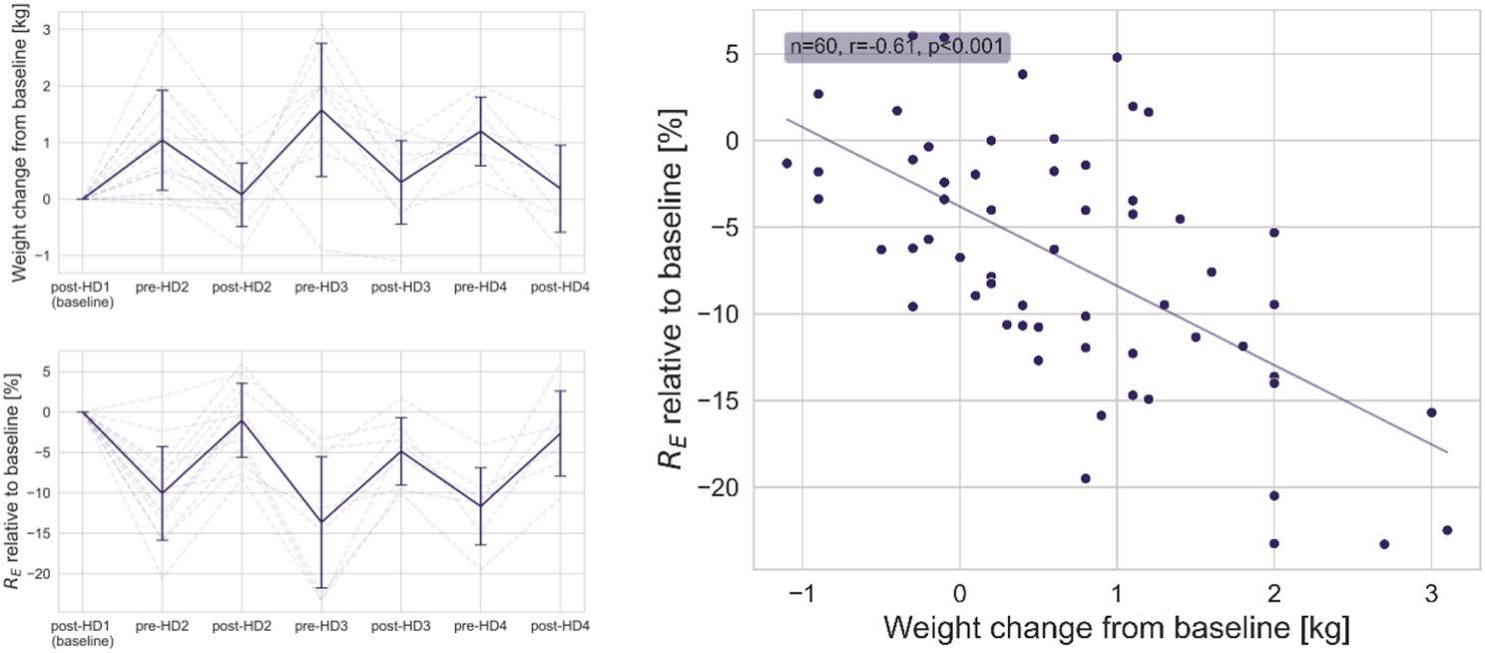
Longitudinal changes in weight [kg] and R_E_ [%] are plotted relative to baseline (upper and lower left, respectively). The time points, denoted as ‘pre’ and ‘post’ for each HD session, are plotted in chronological order for up to three HD sessions (HD2-HD4) and changes are relative to the time point post-HD1 (baseline). The correlation between changes in weight and R_E_for all time points pre-HD2 – post-HD4 is presented to the right

Pearson correlation revealed a moderate negative correlation between the longitudinal changes in R_E_ and body weight (*r* = -0.61 *p* < 0.001; Fig. 5). A similar relationship was found between the % change in R_T_ and weight (*r* = -0.59, *p* < 0.001).

The fluctuations in body weight followed an expected pattern with a higher weight at time points before HD sessions and a lower weight measured at time points after HD sessions.

Changes in R_E_ followed a similar but inversed pattern compared to the body weight, displaying a decreased resistance before HD sessions and increased resistance after HD sessions.

### Cardiovascular changes

Changes in systolic blood pressure were negatively associated with changes in R_E_ (*r* = -0.31, *p* < 0.05), and positively associated with weight changes (*r* = 0.31, *p* < 0.05). Changes in R_T_ did not correlate significantly with changes in systolic blood pressure, and neither the change in weight, R_E_, or R_T_ correlated significantly with changes in diastolic blood pressure.

### Changes in blood parameters

Changes in hematocrit had a weak positive correlation with changes in R_E_ (*r* = 0.32, *p* < 0.05) and R_T_ (*r* = 0.31, *p* < 0.05), and a weak negative correlation with changes in weight (*r* = -0.39, *p* < 0.01). Changes in hemoglobin levels also correlated positively with changes in R_E_ (*r* = 0.30, *p* < 0.05) and negatively with weight changes (*r* = -0.36, *p* < 0.01), but did not correlate significantly with changes in R_T_.

A moderate positive correlation was observed between changes in R_E_ and alterations in the albumin concentration (*r* = 0.51, *p* < 0.001). There was also a moderate but slightly weaker negative correlation between changes in weight and albumin (*r* = -0.44, *p* < 0.001). However, there was no significant correlation between changes in R_T_ and albumin.

Changes in creatinine concentrations was found to have a moderate negative correlation with changes in R_E_ (*r* = -0.62, *p* < 0.001) and R_T_ (*r* = -0.59, *p* < 0.001), and a moderate positive correlation with changes in weight (*r* = 0.59, *p* < 0.001). Alterations in BUN levels were also found to have a negative correlation with changes in R_E_ (*r* = -0.64, *p* < 0.001) and R_T_ (*r* = -0.58, *p* < 0.001), and a strong positive correlation with changes in weight (*r* = 0.71, *p* < 0.001).

### Safety analysis

The patch exposure time was 23–77 h, dependent on the length of the interdialytic period for each patient. Nine of the participants were subject to a single exposure period, and 22 were subject to repeated exposure. Two cases of slight irritation at the patch site after removal were reported. Both instances resolved on their own without the requirement for medical attention. The causality assessment concluded that the reactions were related to the skin adhesive used in the investigational device. One participant experienced a drop in blood pressure (hypotension) during an HD session, which required medical attention by intravenous fluid and oxygen supply. Hypotension is one of the most common side effects of HD and the causality assessment concluded that the event was not related to the investigational device.

## Discussion

The strong positive correlation between total UF volume and the relative change in R_E_ during HD indicates that the device, as hypothesized, is able to measure changes in fluid status during a conventional HD session. The strong relation between decreased fluid volume and increased resistance measured at the upper back was comparable to results previously reported by similar investigations with other bioimpedance devices [17–19]. Although the R_T_ response also had a strong positive correlation with UF volume, the correlation was slightly weaker as compared to R_E_. This is in agreement with previous data, demonstrating that fluid is mainly accumulated in, and drawn from, the extracellular compartment in patients routinely going to HD [20, 21]. Thus, it is expected that changes in fluid status are more accurately reflected by changes in extracellular resistance compared to the total resistance.

Increased concentration of hemoglobin during hemodialysis is an expected effect of hemoconcentration due to fluid removal [22, 23]. The positive correlation between the R_E_ response and the concentration of hemoglobin is thus expected to be due to both variables’ positive association with UF volume. A slight change in the sodium concentration is expected during HD due to slightly different concentrations of sodium in serum and the standard dialysate used. It should however be investigated to what extent changes in electrolyte concentrations affect the measurements in terms of changes in the electrical conductivity of the tissue. Schafetter and colleagues found that total body R_E_ (estimated by cole-cole from 50 frequencies) in fact was affected by both UF volume, postural changes, and diffusion (electrolyte exchange) during HD [24]. UF volume was reported to contribute to 68% of the change in total body R_E_ during a conventional 4 h HD session, while 8% was caused by diffusion and 24% by fluid redistribution due to postural changes. This is in agreement with our findings and indicates that changes in serum sodium may have a slight effect on R_E_, but to a low degree compared to fluid volume.

The investigation did not record the patient’s body posture or postural changes during the hemodialysis (HD) sessions. Given that posture significantly impacts both segmental and whole-body bioimpedance measurements [25, 26], the absence of posture information prevents a comprehensive assessment of its potential influence on the measurements. To mitigate this, we only utilized bioimpedance measurements from time points when the patient was confirmed to be in a seated position—specifically, immediately before and after the HD session.

During HD an increase in R_E_ was associated with a reduction in blood pressure, which was similar to the association between changes in UF volume and systolic blood pressure. In general, patients with fluid overload are expected to experience a decrease in blood pressure during HD as excess fluid is removed. This implies an inverse relationship between UF volume and systolic blood pressure, as observed. However, up to 15% of patients might experience a paradoxical increase in blood pressure during HD [27]. For instance, some patients with heart failure may experience an intradialytic increase in blood pressure due to improved cardiac function. The current investigation included a non-selected population of patients with various comorbidities, and a strong inverse relationship between UF volume and blood pressure would therefore not be expected.

In summary, results obtained during the HD session support that the investigational device is able to track rapid changes in fluid volume in patients with ESRD.

### Longitudinal analysis of fluid status

Short-term changes in body weight offer a reasonable estimation of a patient’s fluid status, particularly in patients with rapid fluctuations in fluid volume. In patients routinely going to HD, the excess weight accumulated compared to a clinically assessed target weight (dry weight) is indeed routinely used to set the UF volume at each HD session [28]. Thus, the significant negative correlation between changes in R_E_ and body weight indicates that the investigational device is able follow longitudinal fluctuations in fluid status as expected based on the intradialytic results. While the relationship between changes in body weight and R_E_ was weaker than the intradialytic correlation between UF volume and R_E_, it is crucial to acknowledge that body weight is susceptible to fluctuations unrelated to fluid status [5, 6] and that it should be a distinct discrimination between the interdialytic weight gain and fluid overload [29]. Further caution is also in order when data is compared over a longer period, as the optimal dry weight will fluctuate over time. It is also widely acknowledged that the process of determining dry weight itself is a complex multifaceted clinical challenge that has inherent inaccuracies, and frequent adjustments are recommended [28].

Hematological changes showed a positive association with R_E_ and a negative association with body weight. This is consistent with the intradialytic correlation between R_E_ and hemoglobin and is most likely driven by hemoconcentration occurring during HD and hemodilution occurring as a result of fluid accumulation between HD sessions [22, 23]. Changes in systolic blood pressure also displayed a similar positive correlation with R_E_ and negative correlation with body weight. For most patients with ESRD, the physician would expect the blood pressure to increase with increased fluid overload [30]. Thus, changes in R_E_ are found to reflect the expected pattern between fluid overload, blood pressure and hemoconcentration to the same extent as changes in weight.

As impaired kidney function compromises the clearance of waste products, including BUN and creatinine, elevated levels of these substances are routinely used as an indicator of renal dysfunction [31]. The negative correlation observed with R_E_ and both waste products could potentially be indirectly driven by fluid accumulation occurring alongside the accumulation of waste products in patients with severely impaired renal function. The fact that similar but positive correlations were observed with changes in weight could support this hypothesis. However, it is important to acknowledge that BUN and creatinine levels are not directly connected to fluid status and residual urine production. Moreover, both BUN and creatinine are confounded by several factors unrelated to kidney function [31–33]. As the primary source of BUN is dietary protein, the concentration is dependent on the nutritional status of the patient. Thus, the strong correlation found between weight and BUN suggests the presence of fluctuations in body weight that are unrelated to changes in fluid balance. This is furthermore in agreement with the fact that R_E_ correlated somewhat weaker with changes in body weight than expected based on the intradialytic correlation with UF volume.

Low level of serum albumin (hypoalbuminemia) is a predictor of mortality in patients with ESRD and is connected to several factors including malnutrition, inflammation, and cardiac disease [34–36]. Findings by Jones et al. have however also indicated an independent inverse relationship between albumin levels and extracellular fluid volume estimated by BIS [37, 38]. This agrees with the positive correlation found between changes in R_E_ and serum albumin levels and negative correlation between albumin and weight.

Absolute measurements of bioimpedance are dictated by body composition including the amount of fat in the measured area. Hence, an interpersonal variation in absolute measurements is expected. Despite this, the relative changes in R_E_ and R_T_ are consistent with the longitudinal fluctuations in body weight and other indirect indicators of fluid status. This supports the use of the investigational device for semi-continuous monitoring of changes in fluid status.

Based on the correlations with changes in body weight, blood pressure and blood sample results, it is concluded that the investigational device follows the relative fluctuations in fluid status over the course of the study period as expected.

### Limitations

As a real-life study, the investigation included a heterogeneous non-selected population of ESRD patients with different levels of disease severity and a range of comorbidities. Several of these comorbidities can importantly also contribute to fluid imbalance regardless of kidney function (e.g. cardiac disease and diabetes). The study was not powered to evaluate the influence of specific comorbidities on the fluid status of the patients, and this represents a limitation of the study.

Another limitation of the study is that many of the patches failed to pass the quality assurance gates, and therefore had to be excluded from the analyses. Based on in-house testing on this specific version, it is likely that water intrusion caused a significant proportion of the erroneous measurements. In a few patches, we also observed some separation between two of the layers in the patch, potentially affecting the contact between the electrodes and the skin. For upcoming studies, the device should be improved with respect to both water resistance and robustness against mechanical stress.

The significance of fluid shifts caused by postural changes was not considered prior to the investigation, and the patient’s body posture and postural changes during HD were not recorded. Thus, it is not possible to accurately assess its influence on our measurements. Lack of comprehensive data on potential confounding factors, in combination with the chosen study design, limits the ability to fully establish the causal relationship between fluid changes and sensor measurements. Future research should incorporate causal inference methodologies and more extensive datasets to clarify these relationships and strengthen the clinical interpretation of the device’s measurements [39].

We also acknowledge that the reference measurements used to assess interdialytic fluid accumulation in this study have their limitations. Longitudinal changes must therefore be interpreted with caution. The ability of the device to detect interdialytic fluid accumulation should be further investigated.

## Conclusions

Currently, there is no gold standard available for feasible, reliable, and affordable assessments of hydration status, and the high costs and patient burden related to recurrent hospitalization of patients with fluid management problems are concerning. Thus, from the perspective of health economics and patient welfare, the successful development of a lightweight non-invasive hydration monitor will indisputably have significant benefits for a wide range of patient populations.

The findings from this investigation indicate that, despite its lightweight and compact design, the investigational device can follow both rapid and gradual changes in fluid volume in patients undergoing regular hemodialysis (HD). The strong correlation observed between the device output and extracted fluid volume during HD supports its capability to measure significant shifts in fluid balance. Additionally, the device demonstrated the ability to track fluctuations in fluid volume over an extended period while being safe to use repeatedly. These results highlight the sensor’s potential within non-invasive longitudinal monitoring of hydration. However, further studies are needed to determine whether the sensor reflects fluid changes in other patient groups, and whether such data can improve patient outcomes.

Given the acknowledged limitations of this investigation, it is relevant to highlight that the investigational device has been subject to continuous development since the completion of the study in 2021. While the current solution has advanced beyond the version used in the present investigation, the results serve as an initial proof of principle for this innovative bioimpedance sensor. Convenient and low-cost solutions have the potential to transform how we monitor patients with fluid management problems, and further studies should explore how the solution, with its most recent advancements, can address existing challenges within fluid management. In summary, the current investigation demonstrates that the wearable bioimpedance sensor, despite its very small size, is able to track changes in fluid balance in patients with ESRD.

## Electronic supplementary material

Below is the link to the electronic supplementary material.

Supplementary Material 1

## Data Availability

The datasets generated in the study are available from the corresponding author upon reasonable request.
