# Supplementary material for: Measuring fluid balance in end-stage renal disease with a wearable bioimpedance sensor
Source: BMC Nephrol. 2025 Jan 8;26:14. doi: 10.1186/s12882-024-03929-9 (PMC11715976; doi:10.1186/s12882-024-03929-9)
Supplement: Supplementary file 1 — Supplementary Material 1 [file 12882_2024_3929_MOESM1_ESM.docx]

# Supplementary Material

## Appendix I

**Table S.1**: Intradialytic change in blood sample analyses (N=46). Significant differences (p<0.05) are indicated (paired t tests, corrected for multiple comparisons). Values are reported as mean ± SD.

| **Analysis** | **Pre dialysis** | **Post dialysis** | **Diff (post-pre)** | **p-value** |
| --- | --- | --- | --- | --- |
| **B-Leukocytes  [10**9/L]** | 6.0  ± 1.5 | 5.7  ± 1.2 | **-0.3  ± 0.7** | **p<0.05** |
| **B-Hemoglobin [g/dL]** | 10.7  ± 1.0 | 10.9  ± 0.8 | **0.2  ± 0.4** | **p<0.05** |
| **B-Thrombocytes [10**9/L]** | 191.6  ± 57.7 | 181.0  ± 44.4 | **-10.6  ± 23.9** | **p<0.05** |
| **PS-Albumin [g/L]** | 36.3  ± 3.2 | 36.7  ± 4.6 | 0.4  ± 1.9 | p=0.51 |
| **P-Glucose [mmol/L]** | 7.8  ± 4.7 | 8.4  ± 2.2 | 0.6  ± 3.1 | p=0.51 |
| **P-Potassium [mmol/L]*** | 5.0  ± 0.9 | 3.5  ± 0.4 | **-1.6  ± 0.9** | **p<0.001** |
| **PS-Blood Urea Nitrogen [mmol/L]** | 18.5  ± 4.5 | 5.6  ± 1.7 | **-12.9  ± 3.2** | **p<0.001** |
| **PS-Creatinine [µmol/L]** | 667.0  ± 169.0 | 236.1  ± 68.6 | **-430.9  ± 115.7** | **p<0.001** |
| **PS-Magnesium [mmol/L]** | 1.0  ± 0.2 | 0.9  ± 0.1 | **-0.1  ± 0.1** | **p<0.001** |
| **PS-Sodium [mmol/L]** | 137.8  ± 2.5 | 137.0  ± 2.0 | **-0.8  ± 2.0** | **p<0.05** |
| **Pt-GFR, estimated [mL/min/1.73m2]** | 6.6  ± 2.4 | 23.5  ± 7.8 | **16.8  ± 6.2** | **p<0.001** |
| **Osmolarity [mosmol/L]**** | 301.9  ± 7.0 | 288.1  ± 5.1 | **-13.9  ± 4.2** | **p<0.001** |

*N=19, samples missing for site B
**Calculated as 2*(PS-Sodium) + P-Glucose + PS-Blood Urea Nitrogen, all in mmol/L
